# Pimarane-type Diterpenes: Antimicrobial Activity against Oral Pathogens

**DOI:** 10.3390/molecules14010191

**Published:** 2009-01-04

**Authors:** Thiago S. Porto, Rander Rangel, Niege A. J. C. Furtado, Tatiane C. de Carvalho, Carlos H. G. Martins, Rodrigo C. S. Veneziani, Fernando B. Da Costa, Adriana H. C. Vinholis, Wilson R. Cunha, Vladimir C. G. Heleno, Sergio R. Ambrosio

**Affiliations:** 1 Núcleo de Pesquisa em Ciências Exatas e Tecnológicas, Universidade de Franca, Franca, SP, Brazil; 2 Departamento de Ciências Farmacêuticas, Faculdade de Ciências Farmacêuticas de Ribeirão Preto, Universidade de São Paulo, Ribeirão Preto, SP, Brazil

**Keywords:** Antimicrobial activity, Pimarane-type diterpenes, Oral pathogens, *Viguiera arenaria*, Structural requirements.

## Abstract

Seven pimarane type-diterpenes re-isolated from *Viguiera arenaria* Baker and two semi-synthetic pimarane derivatives were evaluated *in vitro* against the following main microorganisms responsible for dental caries: *Streptococcus salivarius*, *S. sobrinus*, *S. mutans*, *S. mitis*, *S. sanguinis* and *Lactobacillus casei*. The compounds *ent-*pimara-8(14),15-dien-19-oic acid (PA); *ent*-8(14),15-pimaradien-3b-ol; *ent*-15-pimarene-8b,19-diol; *ent*-8(14),15-pimaradien-3b-acetoxy and the sodium salt derivative of PA were the most active compounds, displaying MIC values ranging from 2 to 8 μg∙mL^-1^. Thus, this class of compounds seems promising as a class of new effective anticariogenic agents. Furthermore, our results also allow us to conclude that minor structural differences among these diterpenes significantly influence their antimicrobial activity, bringing new perspectives to the discovery of new natural compounds that could be employed in the development of oral care products.

## Introduction

Dental caries is a common oral bacterial pathology caused by a biofilm consisting of microorganisms present on the tooth surface [1,2]. It is a disease that has been associated with *Streptococcus* spp., mainly *Streptococcus mutans* and *S. sobrinus*, and *Lactobacillus* spp. [3,4].

Mechanical removal of the dental plaque is the most efficient procedure in caries prevention; however, the majority of the population may not perform this removal efficiently [5]. Moreover, dental treatment is often very expensive and not readily accessible, especially in developing countries [6]. In this sense, extensive efforts have been made toward the search for anticariogenic compounds that can be incorporated into dental products [2, 7] aiming at complementing the mechanical removal of the biofilms from the oral cavity and reducing the incidence of caries in humans [1, 6]. 

Several antibiotics, such as ampicillin, chlorhexidine, sanguinarine, metronidazole, phenolic-antiseptics, and quaternary ammonium-antiseptics, among others, have been very effective in preventing dental caries [3, 8]. However, various adverse effects such as tooth and restoration staining, increasing of calculus formation, diarrhea, and disarrangements of the oral and intestinal flora have been associated with the use of these chemicals [3, 6]. These drawbacks justify the search for new effective anticariogenic compounds that could be employed in caries prevention.

The great diversity of the chemical structures of secondary plant metabolites continues to provide new and important leads against several pharmacological targets [9,10]. Recent studies have demonstrated the great importance of natural products, both plant extracts and isolated compounds, as natural antibacterial agents in oral care products [2,3, 6, 8]. Chung *et al*. [3] have described the specific activity and fast effectiveness of macelignan isolated from *Myristica fragrans* against oral bacteria. Katsura *et al*. [11] have pointed out the great potential of bakuchiol for use in mouthwash for prevention and treatment of dental caries, while More *et al*. [6] have provided insight into the antibacterial properties of the extracts traditionally used in South Africa to prevent and treat of oral problems.

Recently, our research group has demonstrated that kaurenoic acid, a diterpene isolated from *Aspilia foliacea*, could be used as a prototype for the discovery of new effective anti-infection agents against pathogens responsible for caries and periodontal diseases [2]. The study showed that kaurane-type diterpenes can be potentially useful in the development of natural anti-caries and anti-periodontal agents [2]. In view of these results, we have decided to investigate the antimicrobial activity of plant extracts containing other classes of diterpenes. In the present work we describe the effective antimicrobial activity displayed by the dichloromethane root extract from *Viguiera arenaria* (VaDRE) (Asteraceae), as well as the activity of the mainly isolated and two semi-synthetic pimarane-type diterpenes against some oral microorganisms, including *Streptococcus mutans,* which is considered one of the primary causative agents of dental caries [3]. 

## Results and Discussion

**Figure 1:**
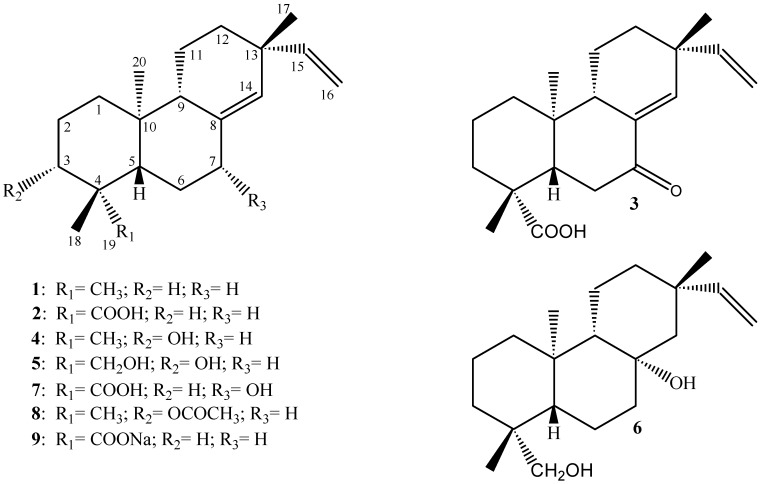
Chemical structures of the pimarane diterpenes from *Viguiera arenaria*.

The chemical structures of the diterpenes studied in this work are presented in Figure 1. The spectral data of all compounds are in agreement with those previously reported in the literature: **1**,**2**, **3**, **6 **and **7** [12,13]; **4** [14]; and **5** [15]. The ^1^H- and ^13^C**-**NMR spectral data analysis of the semi-synthetic derivatives, as well as, comparison with literature [12,13,14] allowed us to establish their structures. 

In this study we demonstrate the significant inhibitory effect that some pimarane-type diterpenes display on the growth of the main microorganisms responsible for dental caries. Among the evaluated metabolites, compounds **2**, **4**, **6**, **8** and **9 **displayed the highest antibacterial activity (Table 1), showing minimum inhibitory concentration (MIC) values lower than 10µg∙mL^-1^ for all dental caries pathogens. Chlorhexidine dihydrochloride was used as positive control, and all MIC values for each microorganism are also described in Table 1. The negative control (4 % DMSO solution) did not affect the growth of the evaluated microorganisms.

According to Ríos and Recio [16], some considerations must be born in mind in the study of antimicrobial assays of plant extracts, essential oils, and compounds isolated from natural sources. These authors have emphasized that MIC values higher than 1 mg∙mL^-1^ for extracts or 0.1 mg∙mL^-1^ for pure metabolites should be avoided, whereas activities in concentrations below 100 μg∙mL^-1^ and 10 μg∙mL^-1^ for extracts and isolated compounds, respectively, are very promising [16]. Based on these criteria the natural diterpenes **2**, **4 **and **6,** as well as the semi-synthetic compounds **8** and **9**, can be classified as potential prototypes for the discovery of new effective anticariogenic agents. Some compounds with potential activity against oral microorganisms have been found in nature. Tsui *et al*. [8] have shown that naringin, a flavonoid that is commonly found in grapefruits, has significant antimicrobial properties against periodontal pathogens *in vitro*. Sanguinarine, an alkaloid isolated from the rhizome of *Sanguinaria canadensis*, has been shown to be a broad spectrum antibacterial agent against various oral bacteria. It has been used as an anticariogenic agent in a wide range of oral care products such as toothpastes and mouthwashes [17], but is industrial application has been dramatically reduced because the use of this compound has been associated with oral leukoplakia [18].

**Table 1:** *In vitro* antibacterial activity (MIC) of the pimarane-type diterpenes against oral pathogens.

Compound	Minimum inhibitory concentration – μg∙mL^-1^ (μM)					
	Microorganism					
	*L. casei*	*S. mitis*	*S. mutans*	*S. sanguinis*	*S. sobrinus*	*S. salivarius*
**VaDRE**	12.0	10.0	12.0	10.0	10.0	10.0
**1**	*	*	*	*	*	*
**2**	3.0 (9.9)	4.0 (13.2)	4.5 (14.9)	2.5 (8.3)	4.0 (13.2)	5.0 (16.5)
**3**	*	*	*	*	*	*
**4**	2.5 (8.7)	4.0 (13.9)	2.5 (8.7)	4.5 (15.6)	6.0 (20.8)	4.0 (13.9)
**5**	*	*	*	*	*	*
**6**	6.0 (19.6)	4.0 (13.1)	6.0 (19.6)	6.0 (19.6)	4.0 (13.1)	3.0 (7.8)
**7**	*	16.0 (50.2)	20.0 (62.8)	*	16.0 (50.2)	*
**8**	6.0 (18.2)	8.0 (24.2)	6.0 (18.2)	6.0 (18.2)	6.0 (18.2)	8.0 (24.2)
**9**	2.0 (6.2)	3.0 (9.3)	2.5 (7.7)	2.5 (7.7)	4.0 (12.3)	3.5 (10.8)
**PC**	0.0922 (0.16)	0.3688 (0.64)	0.0922 (0.16)	0.7375 (1.27)	0.0922 (0.16)	0.0922 (0.16)

* Inactive in the evaluated concentrations (MIC values higher than 80 μg∙mL^-1^); Positive Control (PC) – Chlorhexidine dihydrochloride; Negative control (4 % DMSO solution) did not affect the growth of the microorganisms.

As previously mentioned, our research group has demonstrated that kaurane-type diterpenes can be potentially employed in the further development of natural anti-caries agents. The great importance of understanding the structure-activity relationships of these metabolites has also been pointed out, since minor structural alterations may improve their activities against oral bacteria. Similarly, on the basis of the results presented in Table 1, it is possible to verify that the pimarane-type diterpenes are another important source of secondary plant metabolites with potential for the development of new effective anti-infection agents against microorganisms responsible for caries diseases.

Recently, Urzúa *et al*. [19] have established that a lipophilic decalin ring system, with a strategically positioned hydrogen-bond-donor group (HBD; hydrophilic group), is very important for the antimicrobial activity displayed by diterpenes. Moreover, in this same study the authors pointed out that a second HBD introduced in the decalin ring system led to a reduction in or suppression of the activity. They argued that there were basically two reasons that may explain the reduced antibacterial activity displayed by diterpenoids containing two hydrogen-bond-donor groups: 1) the presence of two HBDs decrease the lipophilicity of the hydrophobic moiety, hindering its interaction with the bacterial membrane; 2) the intramolecular HBD group interactions compete with intermolecular hydrogen-bonds between each HBD and the cell membrane.

**Figure 2:**
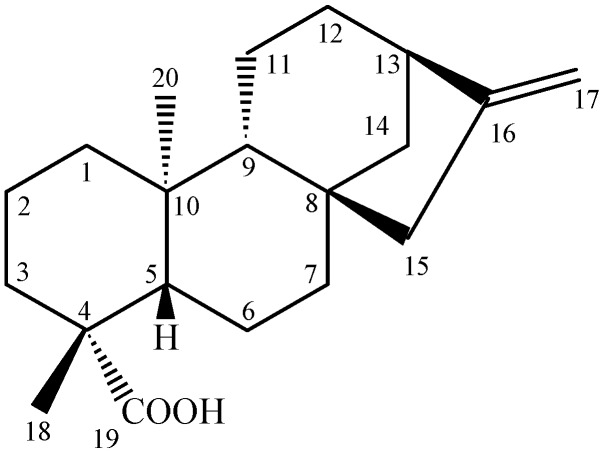
Chemical structure of *ent*-kaur-16(17)-en-19-oic-acid.

A careful observation of the results in Table 1 reveals that compounds **2**, **4**, **6**, **8** and **9,** which contain a HBD at C-3 or C-19, display MIC values much lower than that of compound **1**, which has no HBD in its chemical structure. In fact, the presence of two HBDs in the decalin ring system of compounds **3**, **5** and **7 **really decreases the antimicrobial activity displayed by the pimarane-type diterpenes considerably. In this sense, our results are in complete agreement with those previously reported by Urzúa *et al*. [19]. However, comparison of the MIC values displayed by compound **2** against *Streptococcus salivarius* (16.5 μM), *S. sobrinus* (13.2 μM), *S. mutans* (14.9 μM), *S. mitis* (13.2 μM), *S. sanguinis* (8.3 μM) and *Lactobacillus casei* (9.9 μM) with the values previously described for *ent*-kaur-16(17)-en-19-oic-acid (Figure 2; MIC values higher than 33.06 μM for all oral pathogens) [2] leads to the conclusion that the antimicrobial activity observed for these chemicals is also ruled by other structural factors. These drawbacks highlight the great importance of studying other classes of diterpenes as well as their semi-synthetic derivatives. These studies may help to determine, in a structure-activity context, which other factors contribute to the antimicrobial activity displayed by these metabolites.

Comparing the MIC values of **6** with those of **3**, **5** and **7, **we clearly observe that the proximity between the hydrophilic groups in the latter three significantly reduces their antibacterial activity. This supports the hypothesis of Urzúa *et al*., who describe that the intramolecular interactions between the two HBDs decrease the antimicrobial activity of diterpenes. On the other hand, when the MIC values of **6** and **8** are compared, it is clear that the decrease in lipophilicity and the sucessive decrease in activity, due to the presence of two HBDs, does not work in our case.

## Conclusions

The reports on the antimicrobial activity of natural products against the pathogens responsible for caries diseases are scarce. However, by comparing the MIC values against oral pathogens displayed by some pimarane type-diterpenes described here with MIC values previously published in the literature [2,7,20,21], one can conclude that the pimarane-type diterpenes may be potentially employed in the further development of natural anti-caries agents. Moreover, this work has highlighted the great importance of evaluating other classes of diterpenes as well as their semi-synthetic derivatives, since these studies may help determine the potential of diterpenes as antimicrobial agents. The comparison of the results obtained here with the hypothesis sustained by Úrzua *et al*. [19] establishes the need for a larger number of structures with known activities to totally clarify the structure-activity relationship.

## Experimental

### General

NMR spectra were run on a Bruker DPX 400 spectrometer (400 MHz for ^1^H and 100 MHz for ^13^C). Samples were dissolved in CDCl_3_ or CD_3_OD, and the spectra were calibrated with the solvent signals at 7.26 and 3.30/4.84 (^1^H) or 77.0 and 49.0 (^13^C), respectively; the chemical shifts are given in ppm.

### Plant material

*Viguiera arenaria* Baker (Asteraceae) was collected by Fernando Batista da Costa from the vicinity of the Washington Luís highway (km 223, 22°10 S, 47°59 W, SP, Brazil, in March 1999). The plant material was identified by J. N. Nakajima (Universidade Federal de Uberlândia, MG, Brazil) and E. E. Schilling (University of Tennessee, TN, USA). A voucher specimen (FBC 60) was deposited in the herbarium of the Departamento de Biologia, Faculdade de Filosofia, Ciências e Letras de Ribeirão Preto, Universidade de São Paulo, SP, Brazil, with the code SPFR 4006.

### Extraction and isolation

An aliquot (8.0 g) of the dichloromethane root crude extract from *V. arenaria* (VaDRE), which was prepared in our laboratory in 1999, was suspended in a MeOH/H_2_O solution (9:1 v/v) and partitioned with *n*-hexane and dichloromethane (DCM). The *n*-hexane (2.0 g) and DCM extracts (1.0 g) were re-fractioned using several chromatographic techniques, such as vacuum liquid chromatography, flash chromatography, and preparative thin layer chromatography, as well as recrystallization with methanol, as previously described [22,23]. These procedures furnished 100 mg of *ent*-8(14),15-pimaradiene (**1**); 200 mg of *ent-*pimara-8(14),15-dien-19-oic acid (PA; **2**); 7 mg of 7-keto-*ent*-pimara-8(14),15-dien-19-oic acid (**3**); 100 mg of *ent*-8(14),15-pimaradien-3b-ol (**4**); 20 mg of *ent*-8(14),15-pimaradiene-3b,19-diol (**5**); 5 mg of *ent*-15-pimarene-8b,19-diol (**6**); and 20 mg 7b-hydroxy-*ent*-pimara-8(14),15-dien-19-oic acid (**7**).

### Semi-synthetic derivatives

About 50 mg of **4** were treated with excess acetic anhydride in pyridine according to Da Costa *et al*. [24], to give 37 mg of the C-3 acetoxyl derivate *ent*-8(14),15-pimaradien-3b-acetoxy (**8**). This compound was purified through flash chromatography with *n*-hexane/ethyl acetate (9:1 v/v) as the mobile phase and identified by means of spectrometric analysis and comparison with literature data [14]. ^1^H-NMR: δ 4.52 (1H, dd, *J*=4.2, 11.7 Hz, H-3); 5.14 (1H, s (br), H-14); 5.71 (1H, dd, *J*=10.3, 17.2 Hz, H-15); 4.90 (1H, dd, *J*=2.0, 17.2 Hz, H-16a); 4.95 (1H, dd, *J*=2.0, 10.3 Hz, H-16b); 0.99 (3H, s, H-17); 0.88 (3H, s, H-18); 0.88 (3H, s, H-19); 0.75 (3H, s, H-20); 2.05 (3H, s, H-22 (OCOCH_3_)); ^13^C-NMR: δ 38.3 (C-1); 28.7 (C-2); 81.4 (C-3); 37.2 (C-4); 54.6 (C-5); 21.7 (C-6); 35.9 (C-7); 138.3 (C-8); 51.5 (C-9); 38.4 (C-10); 19.5 (C-11); 36.0 (C-12); 39.0 (C-13); 128.7 (C-14); 147.7 (C-15); 113.3 (C-16); 29.8 (C-17); 22.4 (C-18); 17.3 (C-19); 15.2 (C-20); 171.5 (C-21 (**C**OCH_3_)); 24.5 (C-22 (CO**C**H_3_)).

PA (**2**, 50 mg) was dissolved in *n*-hexane (5 mL) and shaken with a NaOH solution (0.5 mol∙L^-1^, 5 mL) according to Daló *et al*. [25]. The aqueous phase, which contained the sodium salt of PA (**9**) as an emulsion, was filtered in a Büchner funnel, yielding 40 mg of the salt derivative after being washed with cold water. The chemical structure of this compound was established using ^1^H- and ^13^C**-**NMR spectral data analysis and comparison with literature data [12,13]. ^1^H-NMR: δ 5.11 (1H, s (br), H-14); 5.70 (1H, dd, *J*=10.2, 16.3 Hz, H-15); 4.91 (1H, dd, *J*=2.1, 16.3 Hz, H-16a); 4.95 (1H, dd, *J*=2.1, 10.2 Hz, H-16b); 0.96 (3H, s, H-17); 1.15 (3H, s, H-18); 0.71 (3H, s, H-20); ^13^C-NMR: δ 42.1 (C-1); 21.3 (C-2); 40.6 (C-3); 46.9 (C-4); 58.7 (C-5); 26.9 (C-6); 38.0 (C-7); 141.2 (C-8); 53.2 (C-9); 42.1 (C-10); 22.3 (C-11); 38.9 (C-12); 41.4 (C-13); 129.4 (C-14); 149.5 (C-15); 114.5 (C-16); 31.4 (C-17); 31.0 (C-18); 185.2 (C-19); 15.7 (C-20).

### Purity of the evaluated diterpenes

The purity of each diterpene (**1-9 -** Figure 1) was estimated by thin-layer chromatography using different solvent systems. They were also submitted to ^1^H and ^13^C NMR spectral data analysis, which indicated a purity between 95-98% for each compound.

### Antimicrobial assays

The MIC values (the lowest concentration of the compound capable of inhibiting microorganism growth) of the DCM root extract from *V. arenaria* and the pure diterpenes were determined in triplicate using the microdilution broth method [26] in 96-well microplates. Standard strains from the American Type Culture Collection of the following microorganisms were used: *Streptococcus salivarius* (ATCC 25975), *Streptococcus sobrinus* (ATCC 33478), *Streptococcus mutans* (ATCC 25275), *Streptococcus mitis* (ATCC 49456), *Streptococcus sanguinis* (ATCC 10556), and *Lactobacillus casei* (ATCC 11578). The samples were dissolved in DMSO (dimethyl sulfoxide) at 1 mg∙mL^-1^, followed by dilution in tryptic soy broth; concentrations ranging from 80 to 1 mg∙mL^-1^ were achieved. The final DMSO content was 4% (v/v), and this solution was used as negative control. The inoculum was adjusted for each organism, to yield a cell concentration of 5 x 10^5^ colony forming units (CFU)∙mL^-1^. One inoculated well was included, to allow control of the adequacy of the broth for organism growth. One non-inoculated well, free of antimicrobial agent, was also included, to ensure medium sterility. Chlorhexidine dihydrochloride was used as positive control. The microplates (96 - wells) were sealed with plastic film and incubated at 37 °C for 24 h. After that, resazurin (30 mL) in aqueous solution (0.02%) was added to the microplates, to indicate microorganism viability. This procedure was based on the methodology described by Palomino *et al*. [27]. 
